# Exploring the Safety of *Pllans-II* and Antitumoral Potential of Its Recombinant Isoform in Cervical Cancer Therapy

**DOI:** 10.3390/cells12242812

**Published:** 2023-12-10

**Authors:** María José Sevilla-Sánchez, Alejandro Montoya-Gómez, Daniel Osorno-Valencia, Leonel Montealegre-Sánchez, Mildrey Mosquera-Escudero, Eliécer Jiménez-Charris

**Affiliations:** 1 Grupo de Nutrición, Facultad de Salud, Universidad del Valle, Cali 760043, Colombia; maria.sevilla@correounivalle.edu.co (M.J.S.-S.); gabriel.montoya@correounivalle.edu.co (A.M.-G.); osorno.daniel@correounivalle.edu.co (D.O.-V.); leonel.montealegre@correounivalle.edu.co (L.M.-S.); mildrey.mosquera@correounivalle.edu.co (M.M.-E.); 2 Grupo de investigación en Ingeniería Biomédica-GBIO, Universidad Autónoma de Occidente, Cali 760030, Colombia

**Keywords:** bioprospecting, snake venom molecules, PLA_2_, recombinant production, drug discovery, anticancer agents

## Abstract

The antitumor potential of proteins from snake venoms has been studied in recent decades, and evidence has emerged that phospholipases A_2_ can selectively attack cells of various types of tumors. Previous results have shown that phospholipase A_2_ “*Pllans-II*,*”* isolated from *Porthidium lansbergii lansbergii* snake venom, displayed antitumoral activity on cervical cancer and did not alter the viability of non-tumorigenic cells. However, until now, there was no evidence of its safety at the local and systemic levels, nor had experiments been developed to demonstrate that its production using recombinant technology allows us to obtain a molecule with effects similar to those generated by native phospholipase. Thus, we evaluated the impact caused by *Pllans-II* on murine biomodels, determining whether it induced local hemorrhage or increased pro-inflammatory and liver damage markers and histological alterations in the liver and kidneys. Additionally, the protein was produced using recombinant technology using a pET28a expression vector and the BL21 (DE3) *Escherichia coli* strain. Equally, its enzymatic activity and anticancer effect were evaluated on cervical cancer lines such as HeLa and Ca Ski. The results demonstrated that *Pllans-II* did not generate hemorrhagic activity, nor did it increase the pro-inflammatory cytokines IL-6, IL-1B, or TNF-α at doses of 3.28, 1.64, and 0.82 mg/kg. There was also no evidence of organ damage, and only ALT and AST increased in mild levels at the two highest concentrations. Additionally, the recombinant version of *Pllans-II* showed conservation in its catalytic activity and the ability to generate death in HeLa and Ca Ski cells (42% and 23%, respectively). These results demonstrate the innocuity of *Pllans-II* at the lowest dose and constitute an advance in considering a molecule produced using recombinant technology a drug candidate for selective attacks against cervical cancer.

## 1. Introduction

Cancer is a highly prevalent and lethal disease globally, affecting not only the physical health of patients but also their psychological well-being [1]. At the cellular level, this condition is characterized by uncontrolled proliferation, resulting in the reprogramming of metabolism, incessant capacity for replication, resistance to apoptosis, a lack of response to growth inhibitory signals, continuous blood vessel formation, autonomous growth signals, tissue invasion, and metastatic spread [2].

Cervical cancer (CC) is one of the primary contributors to cancer-related mortality in women. Two common CC origin sites are the glandular cells within the endocervix, where the adenocarcinoma usually develops, and the ectocervix (cervix’s superficial layer), where squamous cell carcinoma is produced [3]. Human papillomavirus (HPV) is crucial to developing this disease. Its influence is manifested in inhibiting apoptosis, or programmed cell death, by expressing oncoproteins (E5, E6, and E7) that deactivate the crucial proteins involved in this process [3].

Current treatments for CC often generate side effects or, in some cases, are ineffective [4,5]. The therapeutic procedures used in the fight against cancer have emotional and physical impacts. These side effects include anxiety, depression, mood disturbances, and mental disorders, as well as physical effects such as myelosuppression, cardiac problems, neurological disorders, nephrological complications, cramps, and hair loss [1]. In addition, there is a latent risk of resistance to chemotherapeutic agents and a considerable likelihood of tumor recurrence and the development of metastases, which ultimately limits the effectiveness of treatment [6].

In this sense, bioprospecting has become an area of great interest, via which the sustainable use of natural compounds is sought. Substances such as the secondary metabolites of plants, microorganisms, and biomolecules demonstrate unique mechanisms of action, with more selectivity, and compounds that overcome drug resistance in conventional treatment [7]. Based on the above, in the search for selective bioactive molecules against cancer, snake venom proteins have demonstrated cytotoxicity on several cancer cell lines [8,9,10]. Snake venom phospholipases A_2_s (svPLA_2_s) stand out for their ability to selectively attack tumor cells [9,11,12]. *Pllans-II*, a svPLA_2_s from the northern Colombian *Porthidium lansbergii lansbergii* snake, has exhibited an anticancer effect on HeLa and Ca Ski (cervical cancer cell lines) [13,14], and its partial amino acid sequence was determined using tandem mass spectrophotometry [15]. Research on *Pllans-II* has revealed a concentration-dependent cytotoxic effect associated with cell cycle arrest and apoptotic cell death. Furthermore, *Pllans-II* treatment hindered cell migration and angiogenesis, with minimal impact on non-tumorigenic cells [13,14].

Despite the antitumor potential found for *Pllans-II* and the innocuity evidenced in non-tumoral in vitro models such as the C2C12 (murine skeletal muscle C2C12 myotubes), HUVEC (human umbilical vein endothelial cells), and MCF-10A (non-tumorigenic breast cells) cell lines [9,16], studies are required to verify its innocuous effect on complex physiological systems since some limitations of cell culture models are that they fail to replicate the intricate physiology of the target tissues, and lack the possibility of showing effects on the immune system [17,18]. Although we have obtained evidence in CD-1 strain murine biomodels that *Pllans-II* does not generate myotoxic activity when inoculated into the gastrocnemius muscle, nor lethality via intracerebroventricular injection [16], we still do not have evidence on the effect of *Pllans-II* at the systemic level. Therefore, we evaluated the local and systemic innocuity of *Pllans-II* in murine models based on the determination of local bleeding, pro-inflammatory markers, and histological alterations in the liver and kidneys. Additionally, we produced a recombinant version based on the native *Pllans-II* amino acid sequence and evaluated its cytotoxic effect on HeLa and Ca Ski cell lines.

## 2. Materials and Methods

### 2.1. Snake Venom Collection and Protein Purification

The venom was obtained from specimens of *Porthidium lansbergii lansbergii* in the Atlantic Department, Colombia. Briefly, the snake head was pressurized by a herpetologist against collection tips of 10 μL for venom collection and released after the procedure. The pooled sample was centrifuged to eliminate the cellular content, followed by dehydration using a vacuum centrifuge, and was subsequently preserved at −20 °C until its intended utilization. The study received authorization from the Autoridad Nacional de Licencias Ambientales—ANLA—Colombia via Resolution No. 1070, on 28 August 2015. Additionally, the Ministerio de Ambiente of Colombia awarded a contract for access to genetic resources and their derivative products, numbered 307, on 3 March 2021, to facilitate the development of the current investigation.

The pooled lyophilized venom was reconstituted in a solution of H_2_O/0.1% TFA, and the Pllans-II protein was extracted using RP-HPLC following the previously described method [13]. In summary, two milligrams of venom was separated using a ZORBAX SB-C18 column (250 × 4.6 mm, 5 μm particle diameter; Agilent). Elution occurred at a 1 mL/min rate, employing a gradient from H_2_O/0.1% TFA to acetonitrile/0.1% TFA. The collected samples were monitored at 215 nm using an Agilent 1260 Infinity chromatograph and analyzed using the EZ CHROM ELITE software, version 4.9.005.0 (Agilent Technologies, Inc. 2006, Santa Clara, CA, USA). The specific svPLA_2_ fraction of interest was manually isolated, dehydrated via vacuum centrifugation, and preserved at −20 °C.

### 2.2. Animals

The procedures received approval from the Biomedical Experimental Animal Ethics Committee of Universidad del Valle (CEAS 009-2019). The mice were kept in polypropylene cases under hygienic conditions, housed in groups of 6, adhering to a 12-h light-dark cycle, and provided ad libitum access to both food and water.

### 2.3. Analysis of Pro-Inflammatory Markers and Liver Damage

The mice (twenty in total) were distributed into five experimental groups to evaluate the innocuity of *Pllans-II* at the systemic level via intraperitoneal injection. Three groups received doses of *Pllans-II* (0.82, 1.64, and 3.28 mg/kg), while the positive control group received lipopolysaccharide—LPS (2.5 mg/kg) [19]. The fifth group was treated with PBS as a negative control. The *Pllans-II* doses were selected based on the LD_50_ from *P. lansbergii lansbergii* snake venom, corresponding to 8.2 mg/kg [15]. Due to *Pllans-II* representing approximately 10% of the venom proteome, a value of 10% of the LD_50_ (0.82 mg/kg) was selected, as well as a dose that doubled and quadrupled this value: 1.64 and 3.28 mg/kg, respectively. These doses were chosen to inoculate significant amounts of phospholipase A_2_ to demonstrate possible physiological alterations. Blood samples were collected via cardiac puncture two hours after injection to determine hepatic alterations and the pro-inflammatory response. The samples were coagulated at RT, and serum was obtained via centrifugation at 1500 rpm and 4 °C for 15 min. The serum samples were stored at −20 °C. The concentrations of the pro-inflammatory markers (IL-6, IL-1β, and TNF-α) were analyzed using the ELISA technique, described in Salazar et al. [20] using a microplate reader (IMARK, Bio-Rad, Hercules, CA, USA), and expressed as pg/mL. To determine hepatic alterations, the serum levels of the ALT/GPT, AST/GOT, and ALP enzymes were measured using colorimetric techniques, according to the manufacturer’s recommendations (BioSystems, Barcelona, Spain). The samples were processed in an A-15 analyzer instrument (BioSystems S.A., Barcelona, Spain), and quantifications were expressed as U/L.

### 2.4. Histological Alterations Analysis

The liver and kidneys were extracted and processed following the procedure described by Khalil et al. [21]. In brief, small fractions of the collected tissues were washed with 0.9% saline and then fixed in 10% buffered formalin for 72 h. Afterward, they were washed with ultrapure water for half an hour and dehydrated via one-hour exposure to increasing alcohol concentrations (30, 50, 70, 90, and 100%). Next, the samples were incubated in xylene for two hours and then impregnated thrice in kerosene wax for 45 min each. Once the kerosene solidified, 4–6 μm thick sections were obtained using a microtome mounted on slides. Subsequently, the tissue was deparaffinized in xylene and hydrated via exposure to decreasing alcohol concentrations for one hour. Later, the tissues were stained with hematoxylin and eosin for subsequent microscopic analysis.

Finally, in the analysis of the samples, whole sections of the livers and kidneys of mice exposed to the highest and lowest concentrations were examined (four slides were evaluated for each group). All tissues were examined at 4× to assess the overall tissue appearance, and 10× and 40× magnification was used to identify hemorrhagic areas and alterations in tissue or cellular integrity.

### 2.5. Local Hemorrhage Activity

*Pllans-II* (5 and 10 μg) was dissolved in 100 μL of PBS and administered intradermally into the abdominal region of four mice. Two groups were used as controls: the first group served as a negative control (*n* = 4) using intraperitoneal injection of 100 μL of PBS, and the second group (positive control, *n* = 2) was inoculated with five μg of *P. lansbergii lansbergii* snake venom. After two hours, the animals were sacrificed via dislocation following the methodology described previously [22].

Subsequently, to measure the area and intensity of bleeding, we applied the method proposed by Jenkins et al. [23]. Briefly, the skin was removed, and photographs were taken using a lightbox to minimize the errors generated by surface reflections or the illumination of the samples. Each group of mice was placed in the same lightbox, along with a ruler and a Pantone RGB with the colors 1895 C, 1905 C, 1915 C, 1925 C, and 1935 C. Then, the free software Inkscape 0.91 was used to delimit the hemorrhagic area of the replicas, extracting in parallel the RGB Pantone values and determining the 10 mm scale with the help of the ruler. The contrast between the RGB values of hemorrhage and Pantone allowed for determining the intensity in hemorrhagic units (HaU), while the millimeter scale helped to define the area in mm^2^.

### 2.6. Production, Purification of Recombinant rPllans-II, and Evaluation of Its Activity

The sequence of *Pllans-II* was obtained using mass spectrometry (data not shown) using a Q Exactive Orbitrap instrument (Thermo Fisher Scientific, Waltham, MA, USA) to produce *rPllans-II* using recombinant technology. The sequence was used to construct the insert, bacteria codon optimization was performed, and a PelB signal (to reach the periplasm) and histidine tail were added for protein location and purification. The *E. coli* strain BL21 (DE3) transformed with the pET28a-*rPllans-II* construct was cultured in LB growth medium (200 mL) containing 40 μg/mL kanamycin. The recombinant protein has 123 amino acid residues, 6 histidines in its C-terminal region (for purification), and a PelB signal peptide at the N-terminus. The cell suspension was cultured at 37 °C until it reached an OD_600_ of 0.6. *rPllans-II* was expressed with 1 mM IPTG for 16 h at 16 °C. At the end of induction, cells were harvested via centrifugation (4000 g, 30 min, 4 °C), and the cell pellets were stored at −80 °C. An identical procedure was used to culture the non-induced bacteria as a negative control. The *rPllans-II* expressed in the periplasm was extracted using an osmotic shock method. In summary, the bacterial pellets underwent reconstitution in chilled hypertonic buffer (50 mM Tris-HCl, 20% sucrose, 1 mM EDTA, pH 8.0) and were incubated for 45 min. The resultant mixture was then centrifugated to separate the cells, yielding a supernatant containing periplasmic proteins (fraction 1). Subsequently, the pellet was reconstituted in a cold hypotonic buffer (5 mM MgSO_4_), agitated for 30 min on ice, followed by centrifugation at 9000 g for 10 min at 4 °C. The resulting supernatant (fraction 2) was combined with the previous supernatant to obtain the extracted periplasmic proteins. The soluble protein fraction was purified using an ÄKTA go FPLC system (Cytiva Life Sciences, Marlborough, MA, USA) employing a 1 mL Ni-NTA affinity column (HisTrapHP, Cytiva). Finally, the recombinant protein was desalted in PBS at a pH of 7.4 using HiTrap Desalting columns (Cytiva Life Sciences, Marlborough, MA, USA). The purity of the *rPllans-II* was confirmed using 12% reduced SDS-PAGE and detected using Coomassie blue or silver staining and Western blotting with an anti-His-Tag antibody (enhanced chemiluminescence revelation—ECL). The protein aggregates detected using Western blotting were removed from the *rPllans-II* using a Vivaspin^®^ protein concentrator spin column of 10 kDa (Cytiva Life Sciences, Marlborough, MA, USA).

### 2.7. Determination of Phospholipase Activity

The PLA_2_ activity of the *rPllans-II* was tested using synthetic monodisperse substrate 4-nitro-3-octanoloxybenzoic acid (NOBA), which allows the recognition of PLA_2_ activity via the cleavage of the ester bond in the chromogenic molecule. In summary, following the protocol described by De Arco-Rodríguez et al. [24], 25 μL of various amounts of *rPllans-II* (0.3–5 μg) was mixed in 96-well plates and 200 μL of 10 mM Tris; 10 mM CaCl_2_; 0.1 M NaCl, pH 8.0; and 25 μL NOBA (Thermo Fisher Scientific, Waltham, MA, USA, catalog number J67360) was solubilized in acetonitrile to achieve a NOBA concentration of 0.32 mM. The plates were incubated at 37 °C for 60 min, and the absorbances were recorded at 415 nm using a microplate reader (IMARK, Bio-Rad, Hercules, CA, USA). The phospholipase activity was expressed as the change in absorbance as a function of the quantity of protein (μg). The assay was performed in triplicate.

### 2.8. Cell Cultures

The HeLa and Ca Ski cell lines were purchased from the American Type Culture Collection (ATCC; Manassas, VA, USA) and maintained at 37 °C in an incubator with a humidified atmosphere and 5% CO_2_. EMEM and RPMI 1640 media with 10% FBS, 2 mM L-glutamine, 2 mM sodium pyruvate, 1 mM non-essential amino acids, 100 U/mL penicillin, and 100 mg/mL streptomycin were used for the culture of the HeLa and Ca Ski cell lines, respectively. The cells were split for experiments when they reached 80% confluence.

### 2.9. Cytotoxicity

The cytotoxic activity of *rPllans-II* was evaluated following the method described by Jiménez-Charris et al. [13], with slight modifications. Briefly, 1.5 × 10^4^ HeLa and Ca Ski cells/well were seeded in 96-well microplates for 24 h. Subsequently, the HeLa and Ca ski cells were incubated for 24 h at 37 °C and 5% CO_2_ with the medium in the absence (control cells) or the presence of *rPllans-II* at a concentration of 7.2 μM (IC_50_ for native protein in both cell lines). The IC_50_ for the native *Pllans-II* in both cell lines was determined previously by Jiménez-Charris et al. (2019) [13] and Montoya-Gómez et al. [14] using the Dr Fit software [25]. All assays were performed in triplicate. After treatment, the cells were incubated with MTT for three hours (final concentration 0.5 mg/mL), and the formazan crystals were dissolved by 100 μL of 10% SDS and 0.01 M HCl, followed by incubation for 18 h at 37 °C and 5% CO_2_. The absorbance was measured after 18 h using a microplate reader (Bio-Rad iMark™) at 570 nm. The cytotoxic activity of *rPllans-II* was compared with native PLA_2_ (7.2 μM) and Cisplatin^®^ (332 μM) to determine its efficacy and potential role as an antitumor therapeutic agent. The morphological change in the cells was observed using phase contrast at 20× magnification and an AxioCam ERc 5s Rev. 2.0 (ZEISS, Oberkochen, Baden-Wurttemberg, Germany) adapted to a Primo Vert model trinocular microscope (ZEISS, Oberkochen, Baden-Wurttemberg, Germany).

### 2.10. Statistical Analysis

Significant differences in means and variances were evaluated using one-way ANOVA and a post hoc Tukey test to analyze inflammation and liver damage markers. We used the SPSS Statistics software v24 (IBM 2016) for this analysis. In the cell line experiments, the results were expressed as mean ± SD. The difference between the means of treatments (*rPllans-II* and native *Pllans-II*) and the control was determined using two-way ANOVA, followed by Bonferroni’s post hoc test. The GraphPad Prism v5 software (GraphPad Software, Inc., San Diego, CA, USA) was employed. Statistical significance was indicated by *p* < 0.05.

## 3. Results

### 3.1. Pllans-II Inoculation on Murine Biomodels

#### 3.1.1. Pro-Inflammatory Markers and Liver Damage Analysis

**Figure 1:**
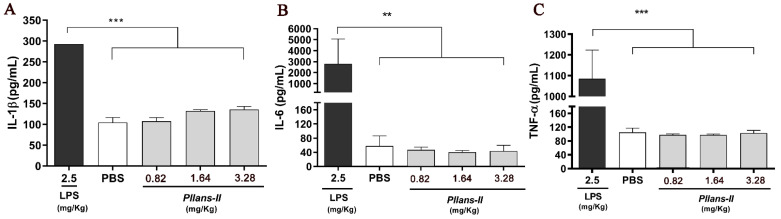
Pro-inflammatory markers were evaluated in mice after two hours of injection of *Pllans-II* (3.28, 1.64, and 0.682 mg/kg) intraperitoneally. (**A**) Interleukin-1 beta (IL-1 β), (**B**) interleukin-6 (IL-6), and (**C**) tumor necrosis factor-alpha (TNF-α) serum level. LPS and PBS were used as positive and negative controls, respectively. Statistically significant with relation to positive control at *p* < 0.05 (** *p* < 0.01, *** *p* < 0.001).

No changes were observed in the levels of interleukin-1 beta (IL-1 β), interleukin-6 (IL-6), or tumor necrosis factor-alpha (TNF-α) after three doses (3.28, 1.64, and 0.82 mg/kg) of injection of *Pllans-II* compared to the negative control (Figure 1A–C). In the case of the commercial pro-inflammatory agent LPS, elevated levels of these cytokines were observed in the plasma (Figure 1A–C). These highly significant differences were evident with a *p*-value < 0.001 for IL-1β and TNF-α and a *p*-value of 0.002 for IL-6.

Additionally, there were no differences in the serum levels of alkaline phosphatase (ALP), a marker of liver damage, after the three *Pllans-II* injection doses. However, there was an increase in the alanine aminotransferase (ALT) serum levels at 1.64 mg/kg dose and in the aspartate aminotransferase (AST) serum levels at the two highest doses (Table 1).

#### 3.1.2. Histological Analysis

**Figure 2:**
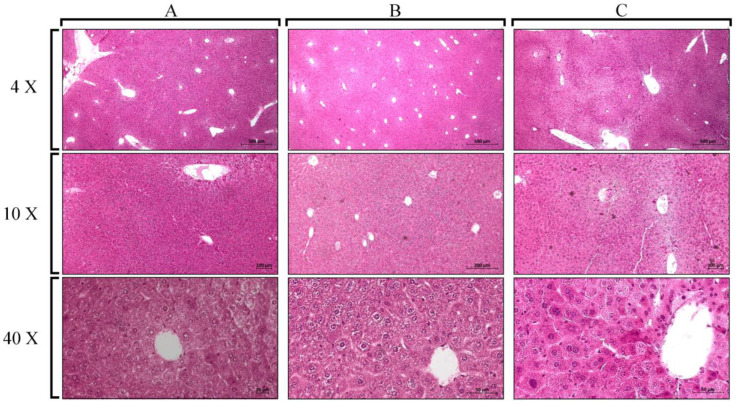
Liver sections of mice inoculated with *Pllans-II* and PBS (negative control) after the staining process with hematoxylin/eosin and visualized under a microscope at different magnifications (4×, 10×, and 40×). (**A**) *Pllans-II* dose of 3.28 mg/kg, (**B**) 0.82 mg/kg, and (**C**) PBS.

**Figure 3:**
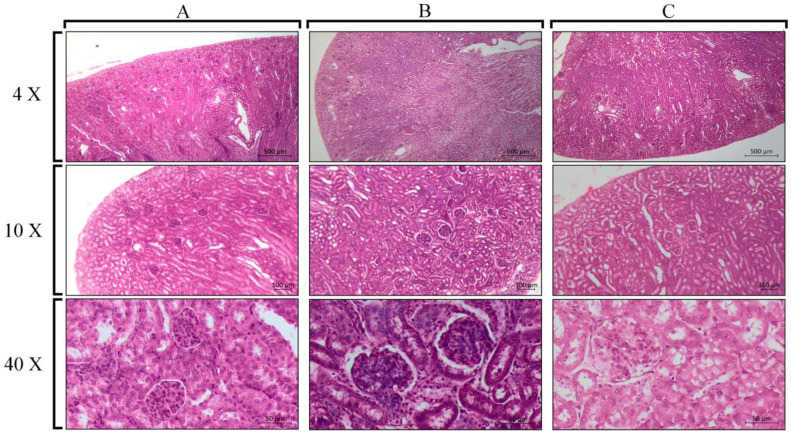
Kidney sections of mice inoculated with two doses of *Pllans-II* and PBS (negative control). The tissues were stained with hematoxylin/eosin and visualized under a microscope at different magnifications (4×, 10×, and 40×). (**A**) *Pllans-II* dose of 3.28 mg/kg, (**B**) 0.82 mg/kg, and (**C**) PBS.

There were non-significant differences in the histological analyses of the liver (Figure 2) and kidneys (Figure 3) of mice inoculated with *Pllans-II* compared with the control group. Hepatic, renal, and glomerular cells maintained their integrity after *Pllans-II* inoculation. Only the highest and lowest *Pllans-II* doses were selected for the organ effect analysis.

#### 3.1.3. Local Hemorrhage

The treatment with *Pllans-II* did not generate hemorrhagic areas for any of the amounts of protein applied (5 µg and 10 µg), similar to the result obtained with the negative control (PBS). In contrast, a significant local hemorrhage was developed for an inoculated amount of 5 µg of the whole venom of *P. lansbergii lansbergii*, with a hemorrhagic area of 2142.46 ± 314.07 mm^2^. *Pllans-II*, like PBS, does not generate blood extravasation. In contrast, treatment with 5 µg of complete venom caused an average of 54.15 ± 21.97 HaU in the biomodels (Supplementary Figure S1).

### 3.2. rPllans-II Production and Evaluation of Its Anticancer Potential

**Figure 4:**
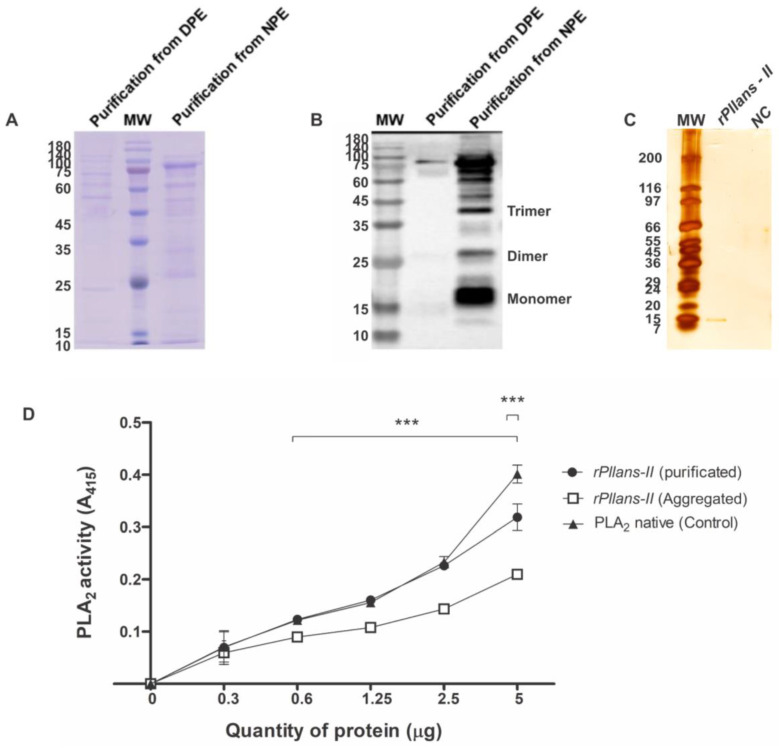
Purification of the *rPllans-II* protein. (**A**) SDS-PAGE profiles of *rPllans-II* under reducing conditions after affinity chromatography. Two μg of native protein extract (NPE) and denatured protein extract (DPE) were loaded into each well. MW showed the molecular weight markers in kDa. (**B**) Western blotting labeled with anti-His-Tag antibody (enhanced chemiluminescence—ECL revelation). The bands at higher molecular weight are protein aggregates. (**C**) SDS-PAGE analysis of soluble *rPllans-II* and negative control (NC—sample from cells without IPTG induction) after 10 kDa Vivaspin^®^ purification. The gel was stained with a silver nitrate solution. (**D**) PLA_2_ activity of purificated and aggregated *rPllans-II*. The native *Pllans-II* was included as a positive control. Each point represents the mean ± SD (*n* = 3) and *** *p* < 0.001.

*rPllans-II* was expressed at low concentrations (5.7 mg protein/L culture medium) and mainly in the denatured protein extract (DPE, Figure 4A,B). Figure 4B shows bands at 15, 30, and 45 kDa, suggesting that *rPllans-II* formed aggregations but was purified after treatment with 10 kDa Vivaspin (Figure 4C). Purified *rPllans-II* exhibited in vitro PLA_2_ catalytic activity similar to the native protein, and the *rPllans-II* aggregates showed a lower PLA_2_ activity (Figure 4D).

**Figure 5:**
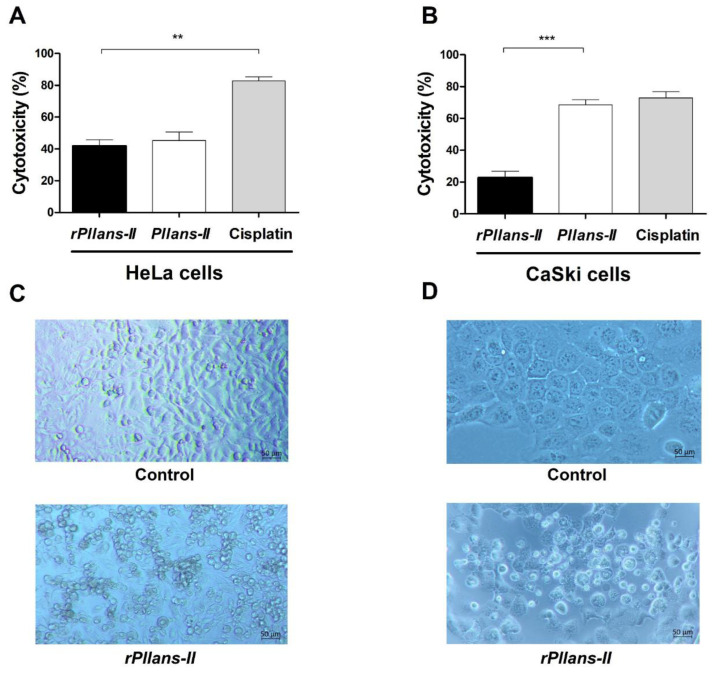
Cytotoxicity and morphological changes induced by *rPllans-II* or *Pllans-II* on cervical cancer cells. (**A**) The adenocarcinoma—HeLa—and (**B**) the epidermoid carcinoma—Ca Ski cells—were seeded in 96-well plates at a density of 1.5 × 10^4^ cells/well and treated with 7.2 μM of *rPllans-II* for 24 h. The MTT assay evaluated the cytotoxicity following the protocol described in the Section 2. Native *Pllans-II* (7.2 μM) and Cisplatin^®^ (332 μM) were positive controls. Bars represent the mean ± SD (n = 2). (**C**) Morphological alterations in HeLa and (**D**) Ca Ski cells after treatment with *rPllans-II*. Note the changes in cell phenotypes to more rounded and clumped appearances. Each cell’s morphologic change was observed under a phase contrast microscope (magnification, 200×). Statistically significant vs. control (Cisplatin^®^): ** *p* < 0.01, or native protein (*Pllans-II*): *** *p* < 0.001.

Interestingly, the recombinant protein alters the viability of both types of cervical cancer cell lines (HeLa and Ca Ski) when tested using the IC_50_ value obtained from the native protein (Figure 5). The most potent cytotoxicity effect was observed against the HeLa cells with a 42% cytotoxicity after 24 h of treatment with 7.2 μM of *rPllans-II* (Figure 5A), which was similar to the cytotoxic effect of the native *Pllans-II*. On the other hand, the cytotoxicity for the Ca Ski cells was lower (23%) (Figure 5B). Both cervical cancer cell lines treated with *rPllans-II* exhibited a rounded morphology with cytoplasmic blebbing, detachment from the plate, and floating in the culture medium. In contrast, the control cells were attached to the plate, displaying a typical morphology. (Figure 5C,D).

However, to validate its pharmacological potential, it must demonstrate that the molecule does not induce local or systemic alterations or generate pro-inflammatory processes that trigger undesirable side effects.

## 4. Discussion

Snake venoms are considered a source of molecules with significant biotechnological potential [26]. Therefore, searching for new therapeutic alternatives against cervical cancer has led us to evaluate one svPLA_2_ named “*Pllans-II*” from the *Porthidium lansbergii lansbergii* snake venom as a possible antitumor pharmacological prototype [13,15,17]. However, to validate its pharmacological potential, it must be demonstrated that the molecule does not induce local or systemic alterations or generate pro-inflammatory processes that trigger undesirable side effects, and our results indicate that the different doses of *Pllans-II* inoculated into the biomodels did not induce an increase in the pro-inflammatory cytokines IL-1β, IL-6, and TNF-α.

Interestingly, in other studies in which the ability of svPLA_2_ to activate immune responses in murine biomodels has been evaluated, an increase in these cytokines has been observed [27,28,29,30,31]. For example, in experiments developed with two svPLA_2_, MT-II, a catalytically inactive Lys-49 variant, and MT-III, an Asp-49 catalytically active enzyme, in addition to the increase in vascular permeability and an influx of leukocytes into the peritoneal cavity in murine biomodels, a time-dependent alteration was evident in the levels of IL-6, IL-1, and TNFα. MT-III elicited a substantial elevation in the IL-1 and IL-6 levels from 180 to 360 min, alongside a notable surge in TNF-α concentrations at the one-hour mark. Conversely, MT-II triggered a significant rise in IL-6 levels exclusively at 180 min and considerably elevated TNF-α concentrations at six hours. These cytokines participate in leukocyte infiltration into the peritoneal cavity and are involved in the inflammatory conditions evidenced in the study [31]. The ability of MT-II to induce an increase in IL-6 levels in the serum of murine biomodels had already been previously demonstrated by Lomonte et al. (1993) [32].

On the other hand, in vitro investigations carried out with promutoxin, an Arg49-type PLA_2_ isolated from *Protobothrops mucrosquamatus* venom, displayed that this protein, devoid of catalytic activity, promotes the release of IL-6, IL-1β, and TNFα in highly purified human monocytes, and the release of IL-6 and TNFα in human T cells, which occurs depending on the concentration of promutoxin applied [33]. The same protein evaluated on murine biomodels was also demonstrated to generate a myotoxic effect, including myoedema, inflammatory cell infiltration, myodegeneration, myonecrosis, and even myolysis [33]. The fact that *Pllans-II* (evaluated at different doses) does not increase the cytokine levels of the inflammatory response, which are altered both in vitro and in vivo when using other svPLA_2_, contributes to the approach of this protein as a selective attack pharmacological prototype against tumor cells, since it is incapable of triggering inflammatory cascades that could lead to damage to organs and tissues. However, our results demonstrate increases in the ALT and AST biomarkers at the two highest doses of *Pllans-II*. Increases in these enzymes in serum are usually related to hepatocellular damage with cytolytic patterns [34,35]. Nevertheless, according to the human diagnostic criteria for increases in aminotransferases, the increase in both enzymes is mild since it is below 2–5× the ULN (upper limit of normal) [36]. These results are consistent with our histological analyses, which did not show alterations in the liver, the main organ in which both enzymes can be released in the event of tissue damage.

Among the requirements for molecules isolated from snake venoms to be considered as potential pharmacological agents are the demonstration of its safety in organs and its inability to generate hemorrhage and trigger pro-inflammatory or suppressive responses of the immune system [37]. Because snake venoms consist of protein complex mixtures such as metalloproteases, svPLA_2_, and other proteolytic enzymes that can induce cytotoxic and hemorrhagic activity, molecules of translational interest must be selected with particular attention to meeting such requirements [38,39]. svPLA_2_ has been implicated in multiple physiological alterations, including hepatotoxicity, nephrotoxicity, and the release of pro-inflammatory cytokines [40,41]. Among svPLA_2_, Lys-49 types are characterized by inducing myotoxic effects [42]. In contrast, the few studies that have evaluated the hemorrhagic activity with svPLA_2_ type Asp-49 have shown that these proteins do not generate hemorrhagic damage on murine biomodels [43,44], which is consistent with the findings of this study since none of the *Pllans-II* amounts caused significant hemorrhagic damage in the skin of the biomodels evaluated.

Although the native protein showed several desirable characteristics as a potential therapeutic agent, large amounts of venom are required to characterize this protein, so we designed a recombinant version of the native protein and evaluated its biological effects on cervical cancer cell lines. This periplasm *E. coli* BL21 (DE3) cell expression strategy was previously used to produce other svPLA_2_s [45,46] or PLA_2_ proteins from different sources [47,48] due to the prokaryotic cell cytoplasm having a reducing environment that prevents the formation of disulfide bonds [49]. The oxidizing conditions and the resident disulfide-bond-forming (Dsb) system of the periplasmic space stimulate proper disulfide bond formation [50,51], allowing the appropriate folding of the mature *rPllans-II* protein with the structural seven disulfide bridges showed by *rPllans-II* conserving the catalytic activity. Additionally, *rPllans-II* displayed a cytotoxic effect similar to its native counterpart, *Pllans-II*, against the HeLa cells. This cytotoxic effect could be related to the C-terminal region of the protein because this svPLA_2_ region plays an essential role in its activity against cell membrane components and could be associated with the affection of tumor cells [52,53,54]. In addition, cervical cancer cells treated with *rPllans-II* exhibited morphological changes that could resemble apoptotic cell death, induced possibly by interactions with integrin-like receptors, as reported for the native *Pllans-II* protein against cervical cancer cells in other studies [13]. The low cytotoxic activity on Ca Ski cells could be related to the *rPllans-II* concentration used. Due to the IC_50_ corresponding to the native protein, it is necessary to develop an experiment to evaluate the *rPllans-II* concentration–response against Ca Ski cells. Probably, membranal components are differentially expressed in Ca Ski and HeLa cells, generating unknown interactions with *rPllans-II* that explain the different cytotoxic effects observed in both cell lines.

## 5. Conclusions

The results demonstrate the safety of *Pllans-II* in murine models at the systemic level, highlighting the low and medium doses (0.82 and 1.64 mg/kg) since no significant increases in pro-inflammatory or hepatic damage were evidenced. Similarly, *Pllans-II* did not generate relevant structural lesions in the liver and kidneys. In addition, the production of its recombinant version showed a conserved catalytic activity represented in its phospholipase activity close to the native protein and a cytotoxic effect on cervical cancer cell lines (HeLa and Ca Ski). Future studies related to the recombinant expression of *Pllans-II* should be directed at the production of larger quantities and the design of peptide derivatives to determine the mechanisms of death induced in a larger panel of cervical cancer cells and its potential as a chemotherapeutic agent.

## Figures and Tables

**Table 1:** Effect on several enzymes’ serum levels after intraperitoneal injection with *Pllans-II* doses in mice.

Biochemical Assay (U/L)	Control	*Pllans-II* (mg/kg)		
	PBS	0.82	1.64	3.28
ALT	33.55 ± 4.15	27.65 ± 3.45	54.45 ± 4.05 ^(a)^	44.55 ± 11.5
AST	131.37 ± 0.35	102.73 ± 7.85	278.75 ± 9.15 ^(b)^	335.53 ± 66.7 ^(c)^
ALP	205.25 ± 4.5	216.5 ± 49.65	205.5 ± 9.33	204.5 ± 3.7

ALT: alanine aminotransferase, AST: aspartate aminotransferase, and ALP: alkaline phosphatase. PBS was used as a negative control. Statistically significant vs. control: ^(a)^,* *p* < 0.05, ^(b)^,** *p* < 0.01, and ^(c)^,*** *p* < 0.001.

## Data Availability

The data presented in this study are available in the article and Supporting Information.

## Supplementary Materials

The following supporting information can be downloaded at: https://www.mdpi.com/article/10.3390/cells12242812/s1, Figure S1: Determination of local hemorrhagic activity after intradermal injection of two doses of *Pllans-II*. (A) An intradermal region without visible lesions after injection with 5 µg or (B) 10 µg of *Pllans-II*. (C) PBS as the negative control. (D) Hemorrhagic areas caused by injection of 5 µg of *P. lansbergii lansbergii* snake venom as a positive control (n = 2). (E) The hemorrhagic units (HaU) corresponding to tissue extravasation are specified in the bar graph. No statistical significance was found, *p* > 0.05. Statistically significant vs. control (Snake venom): *** *p* < 0.001.
