# Supplementary material for: Exploring the Safety of Pllans-II and Antitumoral Potential of Its Recombinant Isoform in Cervical Cancer Therapy
Source: Cells. 2023 Dec 10;12(24):2812. doi: 10.3390/cells12242812 (PMC10741390; doi:10.3390/cells12242812)
Supplement: Supplementary file 1 [file cells-12-02812-s001.zip › cells-2673655-supplementary.pdf]

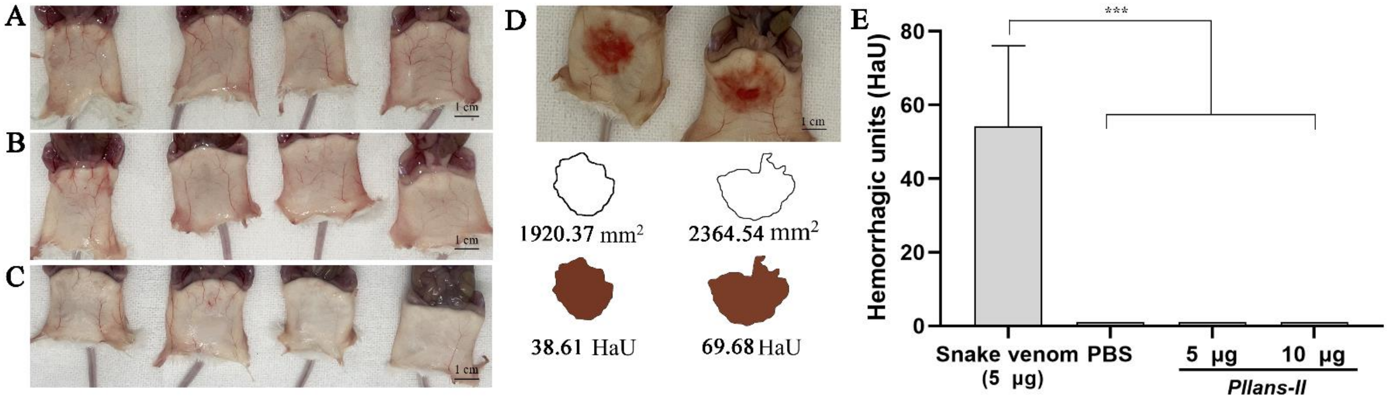

Supplementary Figure S1. Determination of local hemorrhagic activity after intradermal injection of two doses of *Pllans-II*. (**A**) An intradermal region without visible lesions after injection with 5 µg or (**B**) 10 µg of *Pllans-II*. (**C**) PBS as the negative control. (**D)** Hemorrhagic areas caused by injection of 5 µg of *P. lansbergii lansbergii* snake venom as a positive control (n = 2). (**E**) The hemorrhagic units (HaU) corresponding to tissue extravasation are specified in the bar graph. No statistical significance was found, $p > 0.05$. Statistically significant vs. control (Snake venom): *** $p < 0.001$.
